# Supplementary material for: Genomic Landscape and Prediction of Udder Traits in Saanen Dairy Goats
Source: Animals (Basel). 2025 Jan 17;15(2):261. doi: 10.3390/ani15020261 (PMC11759135; doi:10.3390/ani15020261)
Supplement: Supplementary file 1 [file animals-15-00261-s001.zip › animals-3393089-supplementary.pdf]

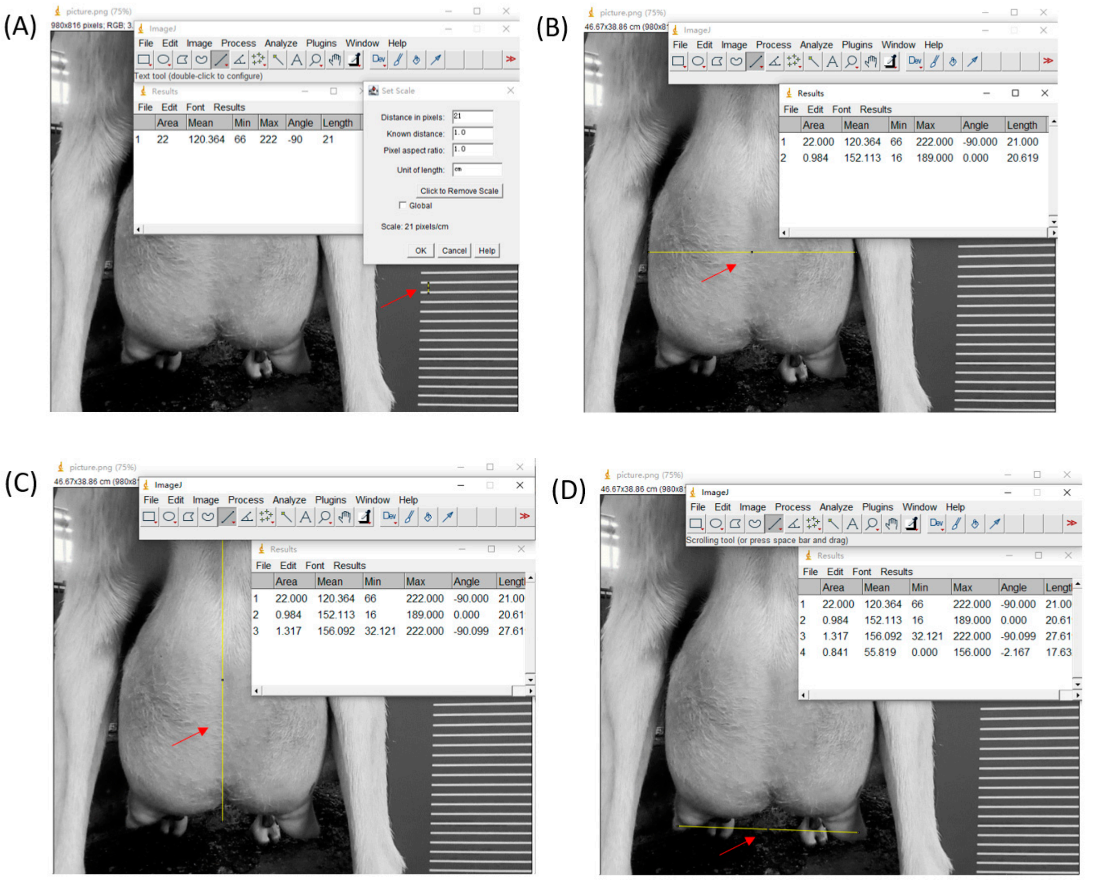


Figure S1. Phenotypic data on udder size in dairy goats were obtained using ImageJ. (A) The scale was calibrated based on the ruler in the image, and measurements of udder width (B), udder depth (C), and teat spacing (D) were performed accordingly.

**Table S1** Genome-wide association signals of udder related traits for Saanen dairy goats

| No. | Traits | Chromosome | Position | Gene name | P value |
|---|---|---|---|---|---|
| 1 | udder width | 3 | 9872130 | *GJB5, GJB4, GJB3, C3H1orf94* | 1.880357e-09 |
| 2 | udder width | 3 | 9717146 | *GJB5, GJB4, GJB3, C3H1orf94* | 2.133195e-07 |
| 3 | udder width | 3 | 65234934 | *PKN2, LOC102184302* | 2.536561e-07 |
| 4 | udder width | 10 | 35231944 | *TBPL2, KTN1* | 5.538103e-07 |
| 5 | udder width | 12 | 73231819 | *DNAJC15, ENOX1* | 5.557494e-07 |
| 6 | udder | 3 | 9867769 | *GJB5, GJB4, GJB3,* | 6.145457e-07 |

| | | | | | |
|---|---|---|---|---|---|
| | width | | | *C3H1orf94* | |
| 7 | udder width | 10 | 35188673 | *KTN1, TBPL2* | 6.939351e-07 |
| 8 | udder width | 7 | 33059408 | *LOC102187402, LOC102187131* | 6.946946e-07 |
| 9 | udder width | 10 | 35190293 | *KTN1, TBPL2* | 7.911481e-07 |
| 10 | udder width | 8 | 61966488 | *ALDH1B1, LOC102184147* | 7.991122e-07 |
| 11 | udder width | 10 | 35179172 | *KTN1, TBPL2* | 8.522974e-07 |
| 12 | udder width | 10 | 35178710 | *KTN1, TBPL2* | 9.013337e-07 |
| 13 | udder depth | 3 | 9872130 | *GJB5, GJB4, GJB3, C3H1orf94* | 1.553601e-09 |
| 14 | udder depth | 3 | 9867769 | *GJB5, GJB4, GJB3, C3H1orf94* | 4.553767e-08 |
| 15 | udder depth | 3 | 9717146 | *GJB5, GJB4, GJB3, C3H1orf94* | 6.582206e-08 |
| 16 | udder depth | 3 | 9595361 | *GJB5, GJB4, GJB3, C3H1orf94* | 9.809573e-08 |
| 17 | udder depth | 3 | 9599893 | *GJB5, GJB4, GJB3, C3H1orf94* | 1.302486e-07 |
| 18 | udder depth | 3 | 9717795 | *GJB5, GJB4, GJB3, C3H1orf94* | 1.547556e-07 |
| 19 | udder depth | 3 | 9602900 | *GJB5, GJB4, GJB3, C3H1orf94* | 1.555806e-07 |
| 20 | udder depth | 3 | 9643984 | *GJB5, GJB4, GJB3, C3H1orf94* | 1.609499e-07 |
| 21 | udder depth | 3 | 9601794 | *GJB5, GJB4, GJB3, C3H1orf94* | 1.771262e-07 |
| 22 | udder depth | 3 | 9858739 | *GJB5, GJB4, GJB3, C3H1orf94* | 2.476634e-07 |
| 23 | udder depth | 3 | 9717026 | *GJB5, GJB4, GJB3, C3H1orf94* | 3.168052e-07 |
| 24 | udder | 3 | 9719781 | *GJB5, GJB4, GJB3,* | 3.168052e-07 |

| | | | | | |
|---|---|---|---|---|---|
| | depth | | | *C3H1orf94* | |
| 25 | udder depth | 3 | 9758206 | *GJB5, GJB4, GJB3, C3H1orf94* | 4.627428e-07 |
| 26 | udder depth | 2 | 123610417 | *LOC102181546, LOC108638390* | 4.681953e-07 |
| 27 | udder depth | 12 | 16063982 | *LOC102179751* | 5.548091e-07 |
| 28 | udder depth | 3 | 12087275 | *GRIK3, ZC3H12A* | 7.990871e-07 |
| 29 | udder depth | 11 | 4619634 | *AFF3, REV1* | 8.395492e-07 |
| 30 | udder depth | 22 | 6201523 | *OSBPL10* | 9.183114e-07 |
| 31 | udder depth | 22 | 6201529 | *OSBPL10* | 9.183114e-07 |
| 32 | teat spacing | 3 | 65319138 | *LOC102184302, PKN2* | 2.400928e-08 |
| 33 | teat spacing | 3 | 65319335 | *LOC102184302, PKN2* | 2.400928e-08 |
| 34 | teat spacing | 3 | 65320137 | *LOC102184302, PKN2* | 2.400928e-08 |
| 35 | teat spacing | 3 | 65320351 | *LOC102184302, PKN2* | 2.400928e-08 |
| 36 | teat spacing | 3 | 65320695 | *LOC102184302, PKN2* | 2.400928e-08 |
| 37 | teat spacing | 3 | 65321299 | *LOC102184302, PKN2* | 2.400928e-08 |
| 38 | teat spacing | 3 | 65321730 | *LOC102184302, PKN2* | 2.400928e-08 |
| 39 | teat spacing | 3 | 65321765 | *LOC102184302, PKN2* | 2.400928e-08 |
| 40 | teat spacing | 3 | 65321823 | *LOC102184302, PKN2* | 2.400928e-08 |
| 41 | teat spacing | 3 | 65322236 | *LOC102184302, PKN2* | 2.400928e-08 |
| 42 | teat | 3 | 65322643 | *LOC102184302, PKN2* | 2.400928e-08 |

| | | | | | |
|---|---|---|---|---|---|
| | spacing | | | | |
| 43 | teat spacing | 3 | 65322859 | *LOC102184302, PKN2* | 2.400928e-08 |
| 44 | teat spacing | 3 | 65325080 | *LOC102184302, PKN2* | 2.400928e-08 |
| 45 | teat spacing | 3 | 65328509 | *LOC102184302, PKN2* | 2.400928e-08 |
| 46 | teat spacing | 3 | 65330532 | *LOC102184302, PKN2* | 2.400928e-08 |
| 47 | teat spacing | 3 | 65332737 | *LOC102184302, PKN2* | 2.400928e-08 |
| 48 | teat spacing | 3 | 65343004 | *LOC102184302, PKN2* | 2.400928e-08 |
| 49 | teat spacing | 3 | 65350338 | *LOC102184302, PKN2* | 2.400928e-08 |
| 50 | teat spacing | 3 | 65350730 | *LOC102184302, PKN2* | 2.400928e-08 |
| 51 | teat spacing | 3 | 65351289 | *LOC102184302, PKN2* | 2.400928e-08 |
| 52 | teat spacing | 3 | 65353133 | *LOC102184302, PKN2* | 2.400928e-08 |
| 53 | teat spacing | 3 | 65354998 | *LOC102184302, PKN2* | 2.400928e-08 |
| 54 | teat spacing | 3 | 65355396 | *LOC102184302, PKN2* | 2.400928e-08 |
| 55 | teat spacing | 3 | 65356411 | *LOC102184302, PKN2* | 2.400928e-08 |
| 56 | teat spacing | 3 | 65356471 | *LOC102184302, PKN2* | 2.400928e-08 |
| 57 | teat spacing | 3 | 65356656 | *LOC102184302, PKN2* | 2.400928e-08 |
| 58 | teat spacing | 3 | 65357566 | *LOC102184302, PKN2* | 2.400928e-08 |
| 59 | teat spacing | 3 | 65357618 | *LOC102184302, PKN2* | 2.400928e-08 |
| 60 | teat | 3 | 65357637 | *LOC102184302, PKN2* | 2.400928e-08 |

| | | | | | |
|---|---|---|---|---|---|
| | spacing | | | | |
| 61 | teat spacing | 3 | 65358300 | *LOC102184302, PKN2* | 2.400928e-08 |
| 62 | teat spacing | 3 | 65358314 | *LOC102184302, PKN2* | 2.400928e-08 |
| 63 | teat spacing | 3 | 65358448 | *LOC102184302, PKN2* | 2.400928e-08 |
| 64 | teat spacing | 3 | 65358460 | *LOC102184302, PKN2* | 2.400928e-08 |
| 65 | teat spacing | 3 | 65358544 | *LOC102184302, PKN2* | 2.400928e-08 |
| 66 | teat spacing | 3 | 65358817 | *LOC102184302, PKN2* | 2.400928e-08 |
| 67 | teat spacing | 3 | 65359435 | *LOC102184302, PKN2* | 2.400928e-08 |
| 68 | teat spacing | 3 | 65359765 | *LOC102184302, PKN2* | 2.400928e-08 |
| 69 | teat spacing | 3 | 65359957 | *LOC102184302, PKN2* | 2.400928e-08 |
| 70 | teat spacing | 3 | 65361025 | *LOC102184302, PKN2* | 2.400928e-08 |
| 71 | teat spacing | 3 | 65361103 | *LOC102184302, PKN2* | 2.400928e-08 |
| 72 | teat spacing | 3 | 65361539 | *LOC102184302, PKN2* | 2.400928e-08 |
| 73 | teat spacing | 3 | 65362210 | *LOC102184302, PKN2* | 2.400928e-08 |
| 74 | teat spacing | 3 | 65362296 | *LOC102184302, PKN2* | 2.400928e-08 |
| 75 | teat spacing | 3 | 65362467 | *LOC102184302, PKN2* | 5.887378e-08 |
| 76 | teat spacing | 3 | 65362664 | *LOC102184302, PKN2* | 5.887378e-08 |
| 77 | teat spacing | 3 | 65367543 | *LOC102184302, PKN2* | 5.887378e-08 |
| 78 | teat | 3 | 65372710 | *LOC102184302, PKN2* | 5.887378e-08 |

| | | | | | |
|---|---|---|---|---|---|
| | spacing | | | | |
| 79 | teat spacing | 3 | 65372839 | *LOC102184302, PKN2* | 5.887378e-08 |
| 80 | teat spacing | 3 | 65156673 | *TRNAE-CUC, LOC102184302* | 6.753169e-08 |
| 81 | teat spacing | 3 | 65373796 | *LOC102184302, PKN2* | 7.338661e-08 |
| 82 | teat spacing | 3 | 65318950 | *LOC102184302, PKN2* | 1.289969e-07 |
| 83 | teat spacing | 3 | 65362466 | *LOC102184302, PKN2* | 2.753863e-07 |
| 84 | teat spacing | 29 | 22854035 | *NELL1* | 2.888431e-07 |
| 85 | teat spacing | 5 | 7961231 | *NAV3, SYT1* | 3.868500e-07 |
| 86 | teat spacing | 3 | 65234934 | *LOC102184302, PKN2* | 4.037464e-07 |
| 87 | teat spacing | 5 | 7961102 | *NAV3, SYT1* | 5.516196e-07 |
| 88 | teat spacing | 3 | 65363136 | *LOC102184302, PKN2* | 6.068067e-07 |
| 89 | teat spacing | 5 | 7961779 | *NAV3, SYT1* | 7.103845e-07 |
| 90 | teat spacing | 5 | 7962115 | *NAV3, SYT1* | 7.293178e-07 |
| 91 | teat spacing | 7 | 96826925 | *ADGRE5, ADGRL1* | 7.458217e-07 |
| 92 | teat spacing | 5 | 7960354 | *NAV3, SYT1* | 7.678510e-07 |
| 93 | teat spacing | 5 | 7960371 | *NAV3, SYT1* | 7.678510e-07 |
| 94 | teat spacing | 5 | 7961522 | *NAV3, SYT1* | 7.969901e-07 |
| 95 | teat spacing | 5 | 7961605 | *NAV3, SYT1* | 7.969901e-07 |
| 96 | teat | 5 | 7961339 | *NAV3, SYT1* | 8.016401e-07 |

| | | | | | |
|---|---|---|---|---|---|
| | spacing | | | | |
| 97 | teat spacing | 5 | 7955985 | *NAV3, SYT1* | 8.663259e-07 |
| 98 | teat spacing | 23 | 2528703 | *DSP, TRNAP-CGG, RIOK1, SNRNP48* | 9.044124e-07 |
| 99 | teat spacing | 5 | 7961619 | *NAV3, SYT1* | 9.144777e-07 |
| 100 | teat spacing | 5 | 7961648 | *NAV3, SYT1* | 9.144777e-07 |
| 101 | teat spacing | 5 | 7959381 | *NAV3, SYT1* | 9.551184e-07 |
